# Selection of M7G-related lncRNAs in kidney renal clear cell carcinoma and their putative diagnostic and prognostic role

**DOI:** 10.1186/s12894-023-01357-9

**Published:** 2023-11-15

**Authors:** Shuangze Zhong, Shangjin Chen, Hansheng Lin, Yuancheng Luo, Jingwei He

**Affiliations:** 1 Guangdong Medical University, Zhanjiang City, 524023 Guangdong Province China; 2 Department of Urology, Yangjiang People’s Hospital affiliated to Guangdong Medical University, Yangjiang, 42 Dongshan Road, Jiangcheng District, Guangdong Province 529500 China

**Keywords:** Kidney renal clear cell carcinoma, N(7)-methylguanosine, lncRNA, Immunotherapy

## Abstract

**Background:**

Kidney renal clear cell carcinoma (KIRC) is a common malignant tumor of the urinary system. This study aims to develop new biomarkers for KIRC and explore the impact of biomarkers on the immunotherapeutic efficacy for KIRC, providing a theoretical basis for the treatment of KIRC patients.

**Methods:**

Transcriptome data for KIRC was obtained from the The Cancer Genome Atlas (TCGA) and International Cancer Genome Consortium (ICGC) databases. Weighted gene co-expression network analysis identified KIRC-related modules of long noncoding RNAs (lncRNAs). Intersection analysis was performed differentially expressed lncRNAs between KIRC and normal control samples, and lncRNAs associated with N(7)-methylguanosine (m7G), resulting in differentially expressed m7G-associated lncRNAs in KIRC patients (DE-m7G-lncRNAs). Machine Learning was employed to select biomarkers for KIRC. The prognostic value of biomarkers and clinical features was evaluated using Kaplan-Meier (K-M) survival analysis, univariate and multivariate Cox regression analysis. A nomogram was constructed based on biomarkers and clinical features, and its efficacy was evaluated using calibration curves and decision curves. Functional enrichment analysis was performed to investigate the functional enrichment of biomarkers. Correlation analysis was conducted to explore the relationship between biomarkers and immune cell infiltration levels and common immune checkpoint in KIRC samples.

**Results:**

By intersecting 575 KIRC-related module lncRNAs, 1773 differentially expressed lncRNAs, and 62 m7G-related lncRNAs, we identified 42 DE-m7G-lncRNAs. Using XGBoost and Boruta algorithms, 8 biomarkers for KIRC were selected. Kaplan-Meier survival analysis showed significant survival differences in KIRC patients with high and low expression of the PTCSC3 and RP11-321G12.1. Univariate and multivariate Cox regression analyses showed that AP000696.2, PTCSC3 and clinical characteristics were independent prognostic factors for patients with KIRC. A nomogram based on these prognostic factors accurately predicted the prognosis of KIRC patients. The biomarkers showed associations with clinical features of KIRC patients, mainly localized in the cytoplasm and related to cytokine-mediated immune response. Furthermore, immune feature analysis demonstrated a significant decrease in immune cell infiltration levels in KIRC samples compared to normal samples, with a negative correlation observed between the biomarkers and most differentially infiltrating immune cells and common immune checkpoints.

**Conclusion:**

In summary, this study discovered eight prognostic biomarkers associated with KIRC patients. These biomarkers showed significant correlations with clinical features, immune cell infiltration, and immune checkpoint expression in KIRC patients, laying a theoretical foundation for the diagnosis and treatment of KIRC.

**Supplementary Information:**

The online version contains supplementary material available at 10.1186/s12894-023-01357-9.

## Introduction

Kidney renal clear cell carcinoma (KIRC) is the most common histological type of renal cell carcinoma (RCC), accounting for more than 80% of all RCC cases, and is characterized by high heterogeneity, invasiveness, and poor prognosis [1]. Currently, partial nephrectomy and radical nephrectomy are the main treatments for early-stage patients [2]. However, early surgical treatment is only effective in some patients, and some local tumor patients may experience recurrence and metastasis after surgery [3, 4], leading to poor prognosis [5]. Clinical studies have shown that RCC is insensitive to adjuvant therapy, radiotherapy, and chemotherapy after surgery, and the recurrence and metastasis rates are almost unaffected [6, 7]. High-dose interleukin-2 has become a first-line treatment option for patients with good Memorial Sloan-Kettering Cancer Center (MSKCC) scores and previously untreated metastatic renal cancer [8]. Immune checkpoint inhibitors have also been used as a first-line treatment option for advanced KIRC [9, 10]. Despite the development of tyrosine kinase inhibitors (TKIs) and immune checkpoint blockers (ICBs), such as Sorafenib, nivolumab, and ipilimumab, there are still reports of tumor recurrence and metastasis after treatment [11, 12]. Therefore, there is an urgent need to explore new biomarkers and potential mechanisms to provide targets for clinical diagnosis and treatment.

M7G modification is a chemical process that adds a methyl group to the nitrogen at the seventh position of guanine in messenger RNA, catalyzed by methyltransferase enzymes [13]. It exists in mRNA, tRNA, rRNA, and microRNA [14], and is one of the most common types of post-transcriptional regulation.

Methylation of the N7 position on guanine in RNA, also known as m7G RNA methylation, plays a crucial role in regulating various fundamental biological processes such as miRNA biogenesis, cell migration, RNA stability, translation, and immunogenicity [15, 16].

Currently known m7G regulatory factors include the Trm8p/Trm82p heterodimer complex and Bud23/Trm112 in yeast, as well as the corresponding orthologs METTL1/WDR4 and WBSCR22/TRMT112 in mammals [13]. Additionally, RNMT and RAM have been shown to participate in m7G modification in mammals [17].

METTL1 and its corresponding cofactor WDR4 form the most comprehensively studied m7G regulatory factor in mammals. The METTL1-WDR4 complex catalyzes m7G modification on various RNA types [18]. Elevated expression of the METTL1-WDR4 complex has been linked to poor prognosis in hepatocellular carcinoma [19], intrahepatic cholangiocarcinoma [20], and lung cancer [21]. As a translation initiation factor, eIF4E plays a crucial role in RNA metabolism by directly binding to the m7G cap and influencing cancer-related mRNA expression at various levels, including nuclear mRNA export, translation, and stability [22, 23]. Studies have shown that eIF4E overexpression is associated with cell proliferation and invasiveness in RCC and negatively correlated with microRNA-15a expression [24]. Inhibiting eIF4E can reduce malignancy and increase sensitivity of RCC cells to chemotherapy and immunotherapy [25]. AGO2, a key element of the RNA-induced silencing complex, can inhibit mRNA translation by binding to the m7G cap [26]. The AGO2 rs4961280 AA/AC genotype is considered a biomarker of poor prognosis in RCC and the expression level of AGO2 can effectively reflect kidney cancer invasiveness [27]. Evidence increasingly suggests that m7G methylation is closely associated with tumor occurrence and development [28–30].

Non-coding RNA (ncRNA) is a type of RNA molecule that does not encode proteins but plays various physiological roles in cells [31, 32]. Long noncoding RNA (lncRNA), as one of the major types of ncRNAs, is characterized by its length exceeding 200 nucleotides [33]. LncRNA has been demonstrated to be an effective regulator of gene expression, achieving this through mechanisms such as chromatin remodeling, transcriptional regulation, post-transcriptional processing, and protein metabolism regulation [34]. Furthermore, lncRNA can exert significant influence on tumor cell proliferation and migration by regulating alternative splicing [35]. SNHG12 enhances tumor progression and sunitinib resistance in RCC by upregulating CDCA3 [36], while MIAT affects patient prognosis by promoting KIRC cell proliferation and metastasis through miR-29c-dependent Loxl2 regulation.Silencing MIAT was found to inhibit in vitro cell proliferation, migration, and invasion, as well as suppress tumor formation in vivo in KIRC according to animal experiments [37]. Therefore, a thorough investigation into the role of lncRNA in KIRC could be of great significance for its treatment.The ENCODE project’s research findings suggest that there may be over 28,000 different lncRNAs encoded in the human genome, many of which are yet to be discovered and annotated [38]. While it remains a challenging task to understand the functions and detailed characterization of all lncRNAs, transcriptome profiling through sequencing analysis has identified thousands of lncRNAs that are abnormally expressed or mutated in different cancers [39]. Recent research has discovered m7G modifications in pri-miRNA and lncRNA using novel m7G detection techniques [40, 41]. Notably, a recent study found that an m7G-related lncRNA risk model holds potential value in predicting tumor prognosis and immunotherapy outcomes [42]. However, the potential of m7G-related lncRNA as a prognostic biomarker for KIRC and predicting immune therapy response remains unclear.

To investigate the prognostic value of m7G-related lncRNAs in KIRC, the lncRNA expression and clinical data of KIRC patients sourced from The Cancer Genome Atlas (TCGA) public database was used to screen biomarkers for KIRC, and validated their prognostic value and predictive significance with clinical features through survival analysis, univariate, and multivariate analysis. Additionally, a column chart model was constructed for personalized prognosis evaluation, and its clinical value was verified using lncRNA expression and clinical data obtained from the International Cancer Genome Consortium (ICGC) as an external dataset. Finally, enrichment analysis was performed to explain the potential mechanisms of KIRC and explore the relationship between biomarkers, immune cells, and immune checkpoint sites.

## Materials and methods

### Data source

Transcriptome data of KIRC was obtained from the The Cancer Genome Atlas (TCGA) database, consisting of 531 KIRC samples and 72 normal control samples, serving as the training set. LncRNAs with zero expression in over 50% of the samples were excluded, resulting in the final TCGA-KIRC-lncRNA expression matrix. The ICGC-RECU-EU dataset, comprising 91 KIRC samples and 45 normal control samples, was downloaded from the International Cancer Genome Consortium (ICGC) database as the validation set. Additionally, 29 m7G-related genes were extracted from published studies [15, 20].

### Weighted gene co-expression network analysis (WGCNA)

First, clustering analysis using the Hclust function was performed on the TCGA-KIRC dataset to identify outlier samples and generate a clustering tree [43]. Network topology analysis was then conducted using the pickSoftThreshold function to determine the soft-thresholding power and assess scale-free topology [44]. The dynamic tree-cutting method was applied to identify co-expression modules, with a minimum module size of 50 for each lncRNA module. Hierarchical clustering trees were plotted, and modules with a correlation above 0.75 were merged based on correlation analysis [43]. Pearson correlation analysis was performed to calculate the correlation coefficient (cor) between each module and the disease. The top two modules with the highest |cor| value were selected as key modules, and the lncRNAs within these modules were considered KIRC-related module lncRNAs [44].

### Identification of differentially expressed m7G-related lncRNAs (DE-m7G-lncRNAs) in KIRC patients

Differential analysis of the lncRNA expression matrix from the TCGA-KIRC dataset was performed using the “DESeq2” R package. The criteria of adj.*p*.value < 0.05 and |log_2_FC| > 1 were used to identify differentially expressed lncRNAs between KIRC samples and normal control samples [45]. The results were visualized using volcano plots generated with the “ggplot2” R package [46]. Heatmaps were also created using the “Pheatmap” R package to display the expression patterns of the top 20 upregulated and downregulated lncRNAs [46].

Pearson correlation analysis was then conducted in the TCGA-KIRC dataset to determine the correlation coefficients between m7G-related genes and all lncRNAs. lncRNAs with |cor| > 0.6 and *p* < 0.001 were considered as m7G-related lncRNAs [47].

The DE-m7G-lncRNAs were obtained by taking the intersection of the KIRC-related module lncRNAs, differentially expressed lncRNAs between KIRC and normal controls, and m7G-related lncRNAs [48]. The expression patterns of DE-m7G-lncRNAs in KIRC and normal control samples were visualized using both volcano plots and heatmaps created with the “ggplot2” and “Pheatmap” R packages, respectively [46].

### Machine learning algorithm for biomarker selection

The “xgboost” R package was used to perform Extreme Gradient Boosting (XGBoost) analysis on the DE-m7G-lncRNAs [49]. The top 10 lncRNAs with the highest importance scores were selected as feature lncRNAs [49].

Simultaneously, the “Boruta” R package was employed to conduct Boruta analysis on the DE-m7G-lncRNAs [50]. The top 10 lncRNAs with the most significant importance were chosen as feature lncRNAs [50] .

The characteristic lncRNAs obtained by XGBoost and Boruta algorithms were intersected to obtain the biomarkers of KIRC [48].

### Evaluation of biomarker prognostic value

The TCGA-KIRC dataset patients were divided into high/low expression groups based on the median expression levels of each biomarker. The relationship between the biomarkers and the survival outcomes of KIRC patients was explored using Kaplan-Meier (K-M) survival analysis [51].

Next, to investigate the prognostic value of the biomarkers and clinical factors, the R package “survival” was used to perform Univariate Cox regression analysis on the biomarkers and clinical factors of TCGA-KIRC dataset patients, including age, gender, grade, stage, T stage, N stage, and M stage. Significant factors associated with KIRC survival were selected [47]. Multivariate Cox regression analysis was then conducted on the identified factors to determine their association with the prognosis of KIRC patients [47]. A nomogram was constructed based on the factors associated with the prognosis of KIRC patients. The accuracy of the diagram was evaluated using calibration curves, and decision curves along with clinical impact curves were used to assess its clinical significance [47].

### Validation of biomarkers

The expression differences of the biomarker lncRNAs between KIRC patients and normal controls were explored in both the TCGA-KIRC dataset and the ICGC validation set. The significance of the differences was assessed using the Wilcoxon test [52]. Pearson correlation analysis was performed in both datasets to investigate the relationship between the biomarkers and clinical features [47]. Furthermore, group analysis was conducted to assess the differences in expression levels of the biomarkers among various subgroups of clinical features [47]. Receiver operating characteristic curves (ROC) were plotted using the “pROC” package in R to evaluate the diagnostic accuracy of the biomarkers for KIRC [47].

### Subcellular localization

The sequence information of the biomarkers was retrieved from the ENSEMBL database, and their subcellular localization was predicted using the lncLocator online tool (http://www.csbio.sjtu.edu.cn/bioinf/lncLocator/#) [53].

### Gene set enrichment analysis (GSEA)

The patients were divided into high and low expression groups based on the median expression values of each biomarker. GSEA was conducted on the biomarkers using the “clusterProfiler” R package and the gene set c2.cp.kegg.v7.5.1.symbols.gmt [54].

### Immune infiltration landscape

Using the single-sample gene set enrichment analysis (ssGSEA) algorithm, the infiltration levels of 28 immune cell types were calculated in the TCGA-KIRC dataset [55]. Pearson correlation analysis was conducted to explore the correlation between the biomarkers and differential immune infiltrating cells [47], as well as the correlation between the biomarkers and common immune checkpoints [47].

## Results

### Identification of module-related lncRNAs associated with KIRC

**Fig. 1:**
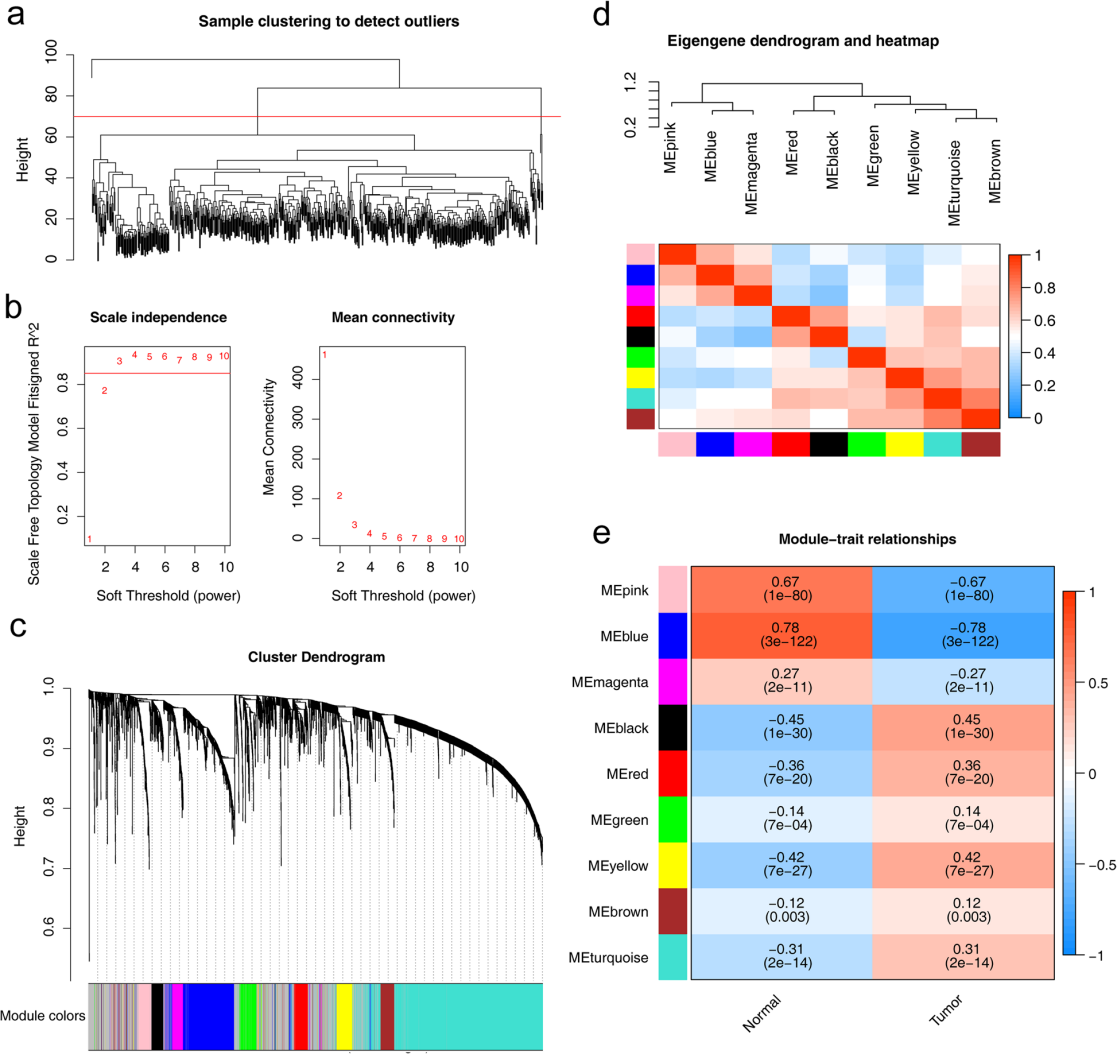
Construction of the co-expression network. **a** Sample clustering tree. **b** Analysis of the network topology of soft threshold power. Soft threshold (power = 3) and scale-free topology fit index (R2 = 0.85). **c** Tree diagram based on hierarchical clustering under optimal soft thresholds. **d** Correlation diagram between modules obtained by clustering according to inter-gene expression levels. **e** Heat map of the correlation between module characteristic genes and clinical features (normal and tumor). Correlations and *p*-values are provided for each module The values in the small cells of the graph represent the two-calculated correlation values cor coefficients between the eigenvalues of each trait and each module as well as the corresponding statistically significant *p*-values. Color corresponds to the size of the correlation; the darker the red, the more positive the correlation; the darker the green, the more negative the correlation

Clustering analysis identified five outlier samples in the TCGA-KIRC dataset, which were subsequently excluded (above the red line) (Fig. 1a). Network topology analysis revealed a scale-free topology with a fitting index of 0.85 when the soft threshold power was set to 3 (red line) (Fig. 1b). Dynamic tree cutting module identification resulted in the identification of nine co-expression modules, with inter-module correlations below 0.75 (Fig. 1c-d). Pearson correlation analysis highlighted the MEblue and MEpink modules as having the highest correlation with KIRC (|cor|>0.5, *p* < 0.05). Therefore, the MEblue and MEpink modules, which consisted of 575 lncRNAs, were selected as module-related lncRNAs associated with KIRC (Fig. 1e).

### Identification of DE-m7G-lncRNAs

**Fig. 2:**
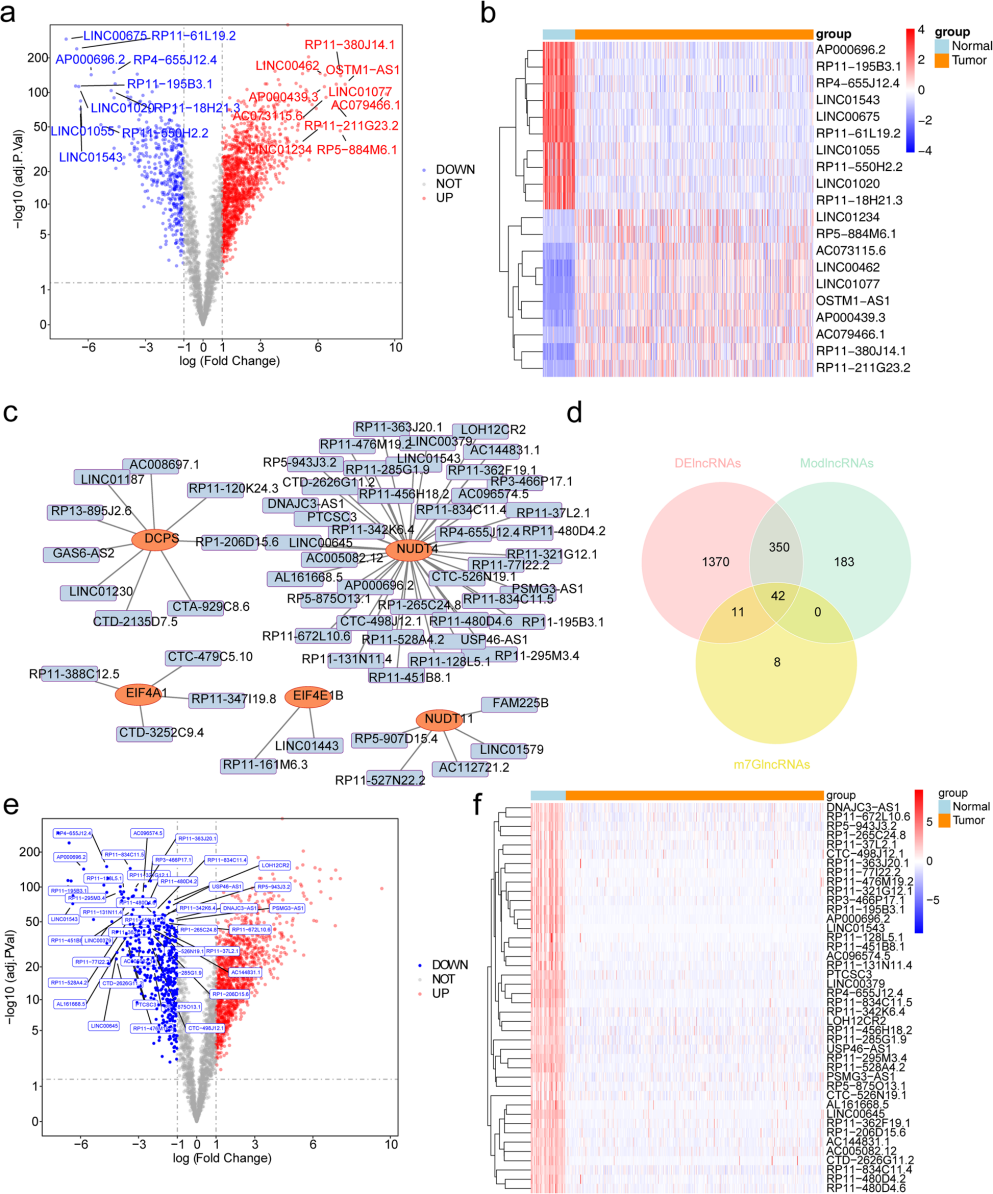
Identification of differentially expressed genes (DEGs). **a**,**b** The volcano plot (**a**) and heatmap (**b**) of differentially expressed lncRNAs between KIRC samples and adjacent normal samples. Red denotes upregulated genes, and blue denotes downregulated genes in both volcano plots and heatmaps. The horizontal axis of the heatmaps represents the samples, and the vertical axis of heatmaps presents the top forty DEGs. **c** Correlation analysis of m7G-associated genes with lncRNAs. **d** The venn plot of KIRC-related modules lncRNAs (yellow), differentially expressed lncRNAs (pink) between KIRC and normal controls, and m7G-associated lncRNAs (green). **e** The volcano plot of 42 differentially expressed DE-m7G-realted lncRNAs. **f** Heat map for the expression of 42 DE-m7G-lncRNAs in normal kidney tissue and tumor tissue

In the TCGA-KIRC dataset, lncRNAs with differential expression between KIRC and normal control samples were screened. Differential analysis of the lncRNA expression matrix identified 1773 lncRNAs that exhibited differential expression between KIRC and normal control samples. Among them, 1342 lncRNAs were significantly upregulated, while 431 lncRNAs were significantly downregulated (Fig. 2a, b).

Correlation analysis further identified 62 m7G-related lncRNAs in the TCGA-KIRC dataset **(**Fig. 2c**)**. By taking the intersection of the module-related lncRNAs associated with KIRC, the differentially expressed lncRNAs between KIRC and normal control samples, and the m7G-related lncRNAs, a set of 42 DE-m7G-lncRNAs was obtained (Fig. 2d). Interestingly, all 42 DE-m7G-lncRNAs were found to be downregulated in KIRC (Fig. 2e, f).

### Identification of biomarkers for KIRC

**Fig. 3:**
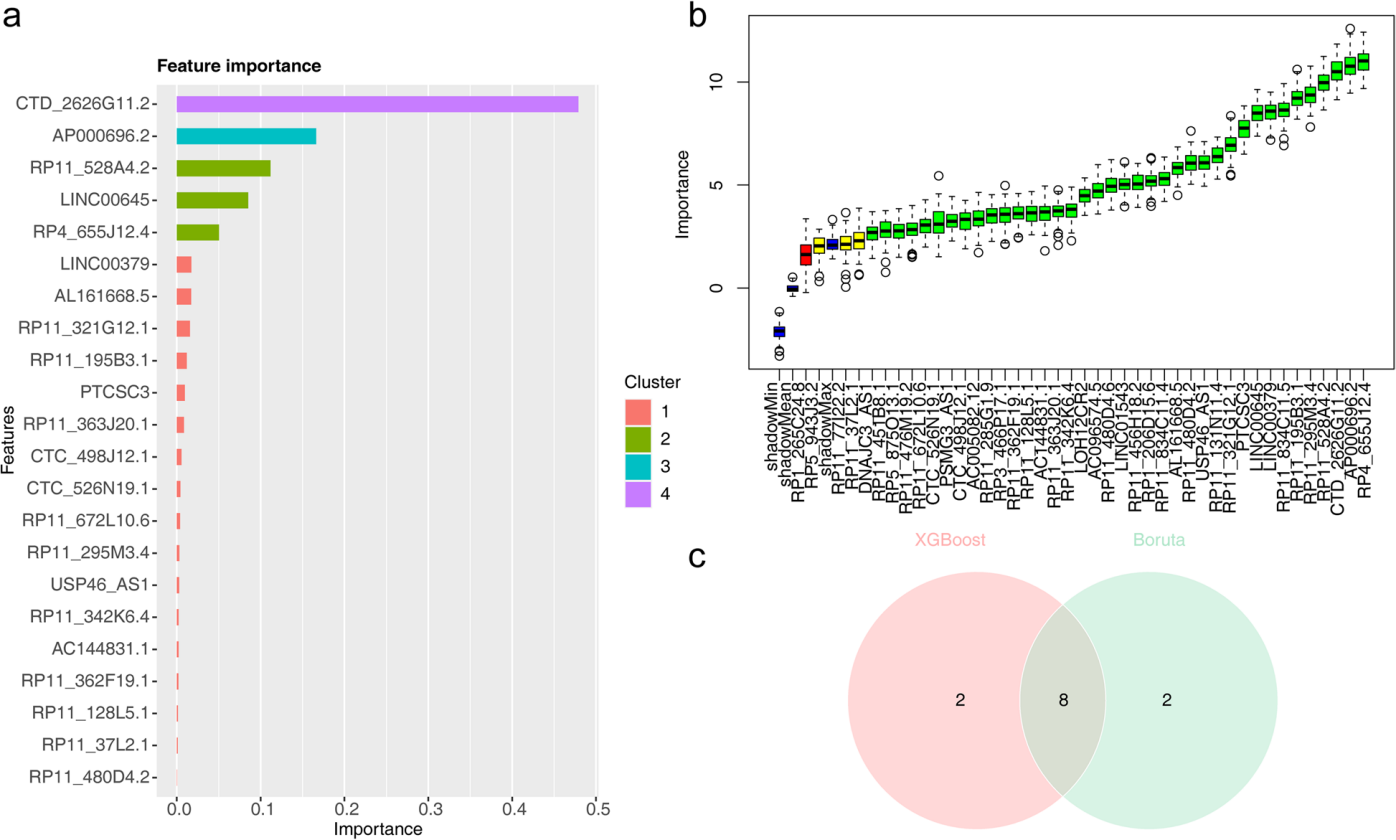
Identification of biomarkers. **a** Importance scores of the features obtained from the XGBoost model trained with the DE-m7G-lncRNAs. Features are listed in descending order of their importance scores, and only the top 10 features are shown in the figure. **b** Feature selection analyzed by Boruta algorithm. The horizontal axis is the name of each variable, and the vertical axis is the Z-value of each variable. The box plot shows the Z-value of each variable in the model calculation. The green boxes represent the 38 important variables, the yellow represents tentative attributes, and the red represents unimportant variables. **c** Venn diagram displaying the intersection results of the top 10 feature lncRNAs obtained from the Boruta and XGBoost algorithms

The intersection of the top 10 feature lncRNAs obtained from both the XGBoost and Boruta algorithms resulted in eight intersecting lncRNAs, which were identified as biomarkers for KIRC **(**Fig. 3a-c**)**. The eight biomarkers were: CTD-2626G11.2, AP000696.2, RP11-528A4.2, LINC00645, RP4-655J12.4, RP11-321G12.1, RP11-195B3.1, and PTCSC3.

### Prognostic prediction of KIRC patients based on biomarker-constructed nomogram

**Fig. 4:**
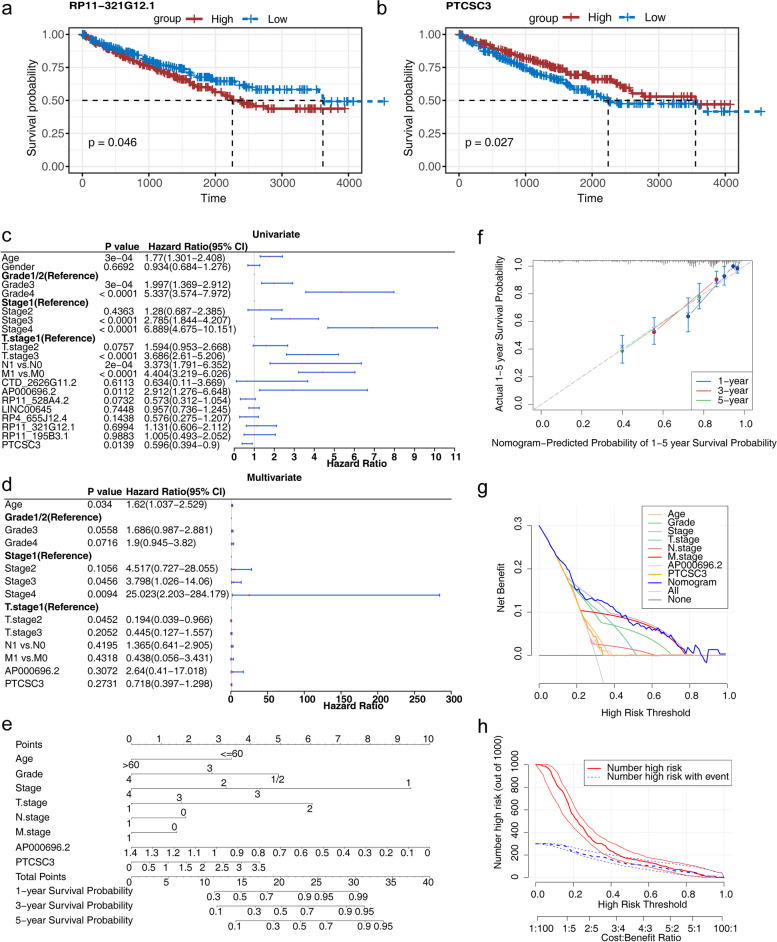
Independent prognostic analysis. **a** Patients with high expression of RP11-321G12.1 have reduced viability. **b** Reduced viability in patients with low PTCSC3 expression. **c** Univariate Cox regression analysis. **d** Multivariate Cox regression analysis. Forest plots show significant urvival-related factors based on univariate Cox regression analysis and multivariate Cox regression analyses. Note: *P* values were calculated by log-rank test. HR: hazard ratio; CI: confidence interval; ∗: *P* < 0.05; ∗∗: *P* < 0.01. **e** Nomogram combining clinicopathological variables and risk score. (f) Calibration curves of the nomogram for predicting survival at 1, 3, and 5 years. The nomogram prediction accuracy is higher if the actual curve is closer to the ideal curve. The gray dotted lines represent the ideal curve, and the blue, red, and green lines represent the actual curves for 1-year, 3-year, and 5-year survival rates. **g** Decision curve analysis (DCA) showing the clinical benefits of a predictive nomogram. **h** Clinical impact curve of nomogram. CIC visually shows that nomogram has high clinical net benefit and confirms the clinical value of nomogram

K-M survival analysis revealed a significant difference in the survival capacity between high and low expression groups of biomarkers PTCSC3 and RP11-321G12.1 in the TCGA-KIRC dataset (*p* < 0.05) (Fig. 4a, b and Figure S1 a-f).

Furthermore, in the TCGA-KIRC dataset, univariate Cox regression analysis showed that age, grade, stage, T stage, M stage, N stage, AP000696.2, and PTCSC3 were associated with the survival capacity of KIRC patients (*p* < 0.05) (Fig. 4c). Multivariate Cox regression analysis indicated that age, stage, and T stage were independent prognostic factors for KIRC patients (*p* < 0.05) (Fig. 4d). A nomogram was constructed based on the factors age, grade, stage, T stage, M stage, N stage, AP000696.2, and PTCSC3, which were associated with the survival capacity of KIRC patients (Fig. 4e). The calibration curve demonstrated the accurate prognostic prediction ability of the forest plot model for KIRC patients (Fig. 4f). Moreover, the decision curve analysis (DCA) curve showed that the forest plot model combining clinical factors and biomarkers had a higher net benefit than individual factors (Fig. 4g). The clinical impact curve (CIC) demonstrated the accurate prognostic prediction ability of the forest plot model for KIRC patients (Fig. 4h).

### Validation of biomarkers

**Fig. 5:**
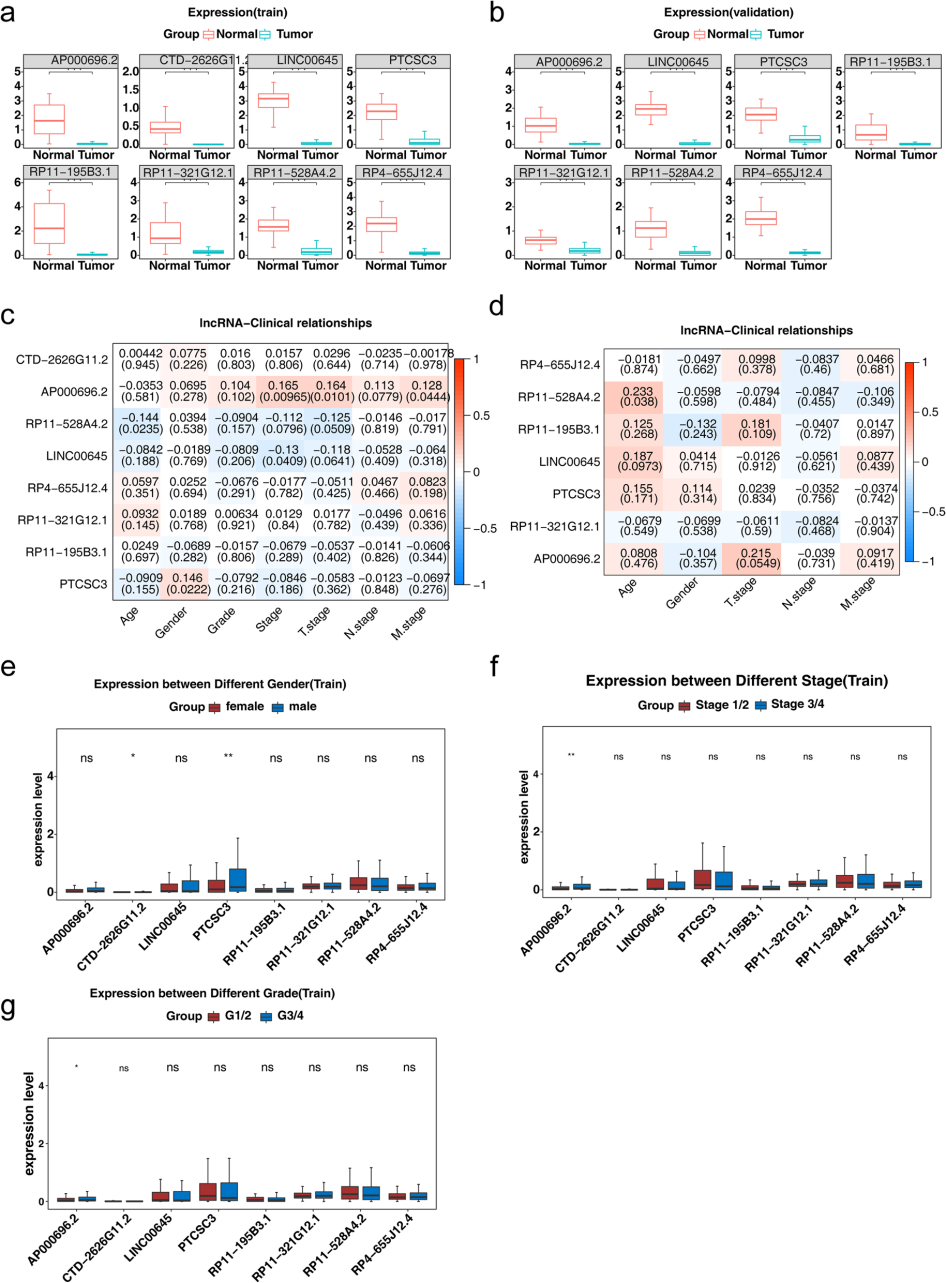
Analysis of expression and clinical correlation for biomarkers. **a**,**b** Differential expression of 8 biomarkers in normal and KIRC tissues in the TCGA-KIRC dataset (training set) (**a**) and the ICGC-RECU-EU dataset (validation set) (**b**). Note: CTD-2626G11.2 was not annotated in the ICGC test set. **c**,**d** Correlation of biomarkers with clinical features in the training set (**c**) and validation set (**d**). **e**-**g** The expression of biomarkers in KIRC patients under different clinical features (including age, tumor grade, tumor stage and tumor T stage) in the TCGA-KIRC dataset

The expression profiles of biomarker were explored in both the TCGA-KIRC dataset and the ICGC-RECU-EU validation dataset. The results revealed that the expression levels of the eight biomarkers in KIRC patients from the TCGA-KIRC dataset were significantly lower than those in normal control samples (p.adj < 0.05) (Fig. 5a and Fig. 2e, f). In the ICGC-RECU-EU validation dataset, the expression of seven biomarkers (including AP000696.2, RP11-528A4.2, LINC00645, RP4-655J12.4, RP11-321G12.1, RP11-195B3.1, and PTCSC3) was detected, and their expression levels were significantly lower in KIRC patients compared to normal controls (p.adj < 0.05) (Fig. 5b).

Correlation analysis in the TCGA-KIRC dataset revealed that RP11-528A4.2 was negatively correlated with patient age, LINC00645 was negatively correlated with tumor stage, PTCSC3 was positively correlated with gender, and AP000696.2 was positively correlated with tumor stage, T stage, and M stage (Fig. 5c).

Furthermore, subgroup analysis revealed significant differences in the expression levels of CTD-2626G11.2 and PTCSC3 between different genders in the TCGA-KIRC dataset (*p* < 0.05) (Fig. 5d). The expression level of AP000696.2 was significantly higher in high-grade KIRC patients compared to low-grade KIRC patients (Fig. 5e). Moreover, the expression level of AP000696.2 was significantly higher in advanced-stage KIRC patients compared to early-stage KIRC patients (Fig. 5f). Additionally, T3/4 stage KIRC patients exhibited significantly higher expression of AP000696.2 compared to T1/2 stage KIRC patients (Fig. 5g). There were no significant differences in the expression levels of the remaining biomarkers among the subgroups based on pathological features (Figure S2a-c).

Furthermore, in both the TCGA-KIRC dataset and the validation dataset, the ROC curve analysis revealed that the area under the curve (AUC) for the biomarkers was greater than 0.95, indicating that these biomarkers can accurately diagnose KIRC (Figure S3a, b).

### Subcellular localization

**Fig. 6:**
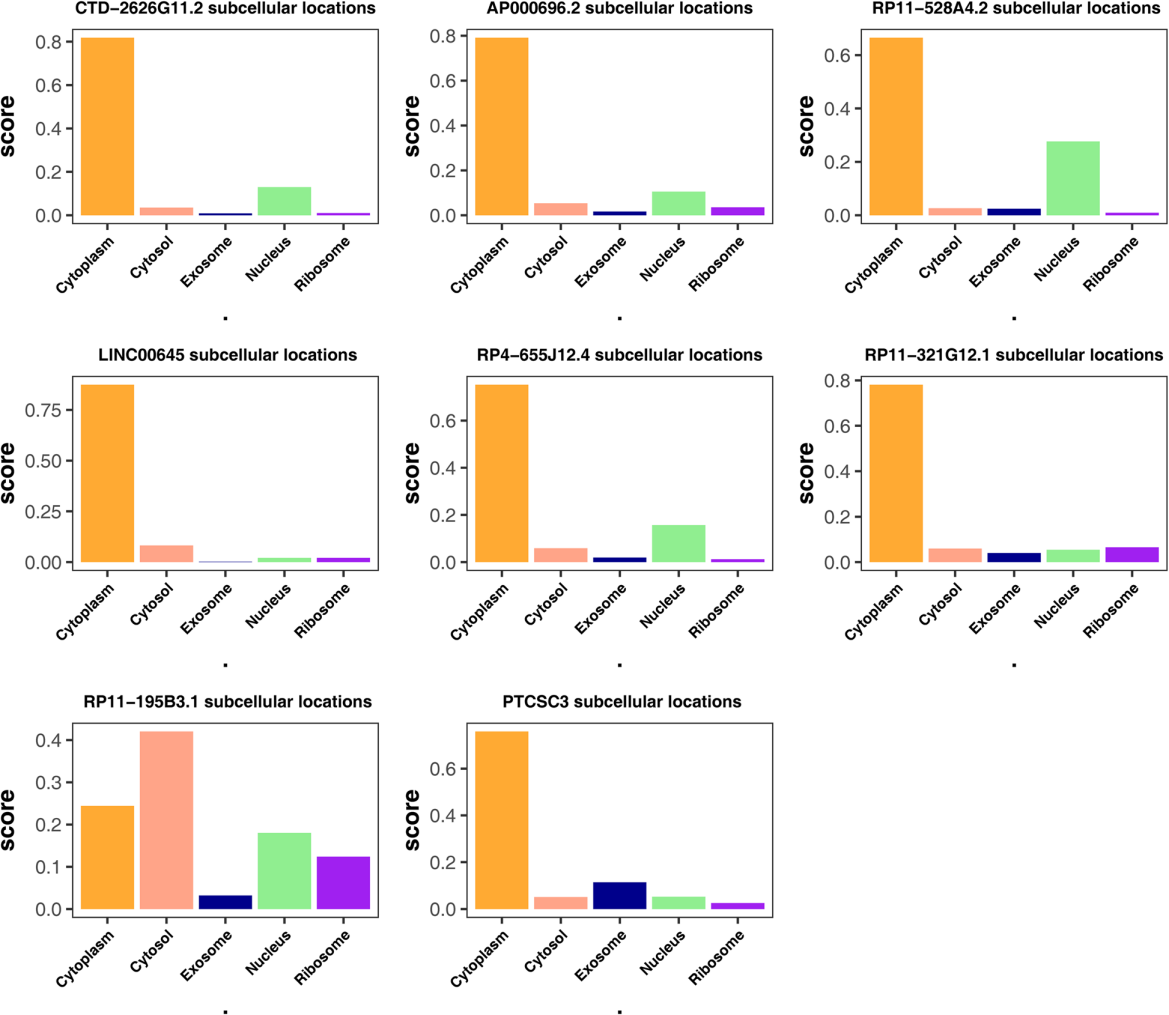
Subcellular localization prediction of 8 biomarkers

Subcellular localization of lncRNAs is closely related to their functions. Subcellular localization prediction was performed for each biomarker. The results revealed that the biomarkers CTD-2626G11.2, AP000696.2, RP11-528A4.2, LINC00645, RP4-655J12.4, RP11-321G12.1, and PTCSC3 were primarily located in the cytoplasm, suggesting their potential involvement in post-transcriptional regulation (Fig. 6 ).

### Biomarkers mainly associated with cytokine-mediated immune response

**Fig. 7:**
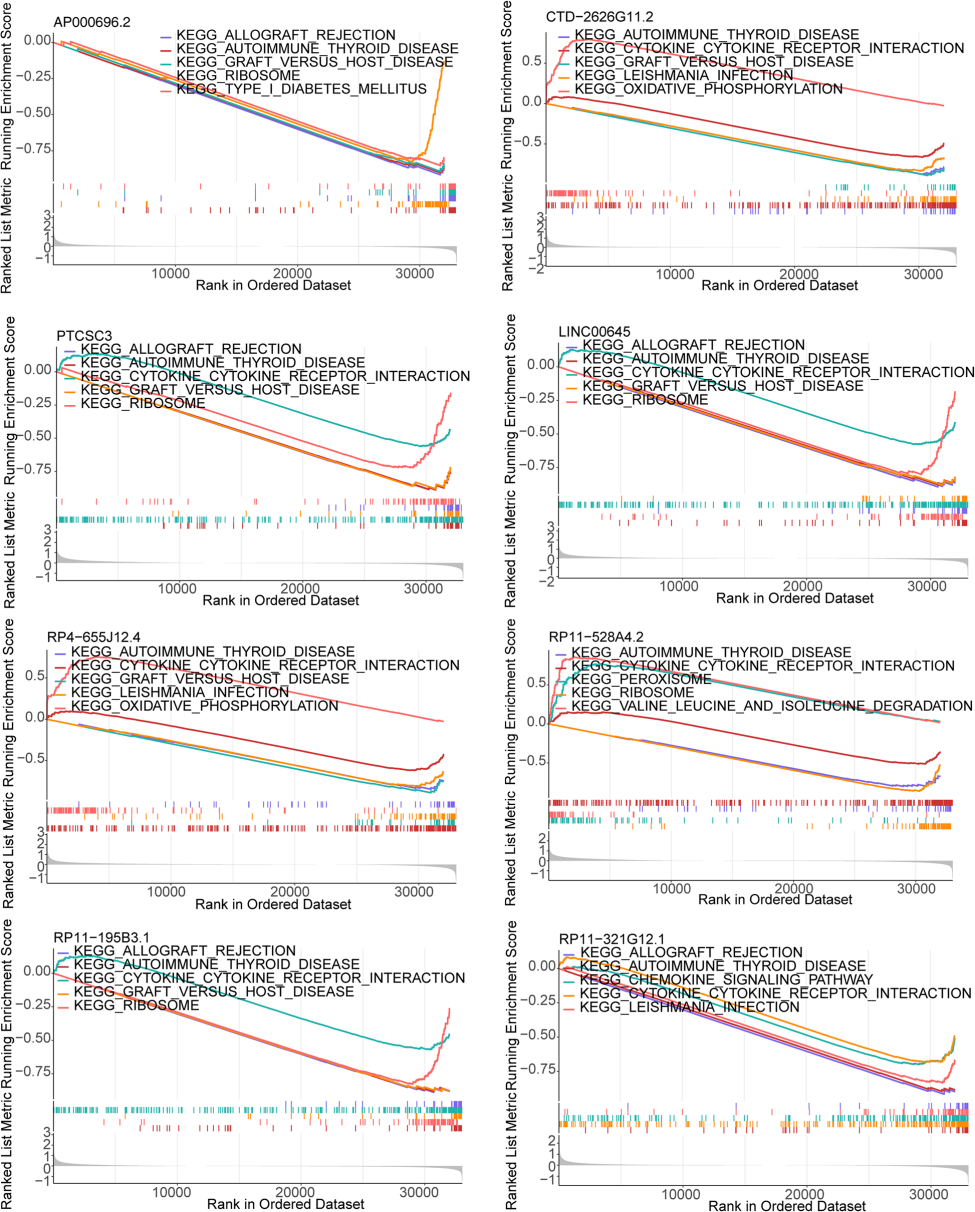
Gene enrichment analysis of different biomarkers

GSEA enrichment analysis revealed that the eight biomarkers were primarily associated with various cellular processes related to immune responses mediated by cytokines. These processes include allograft rejection, autoimmune thyroid disease, graft-versus-host disease, ribosomes, type 1 diabetes, interaction between cytokines and cytokine receptors, leishmaniasis infection, oxidative phosphorylation, chemokine signaling pathway, peroxisomes, degradation of valine, leucine, and isoleucine (Fig. 7).

### The infiltration levels of immune cells in KIRC samples were significantly elevated

**Fig. 8:**
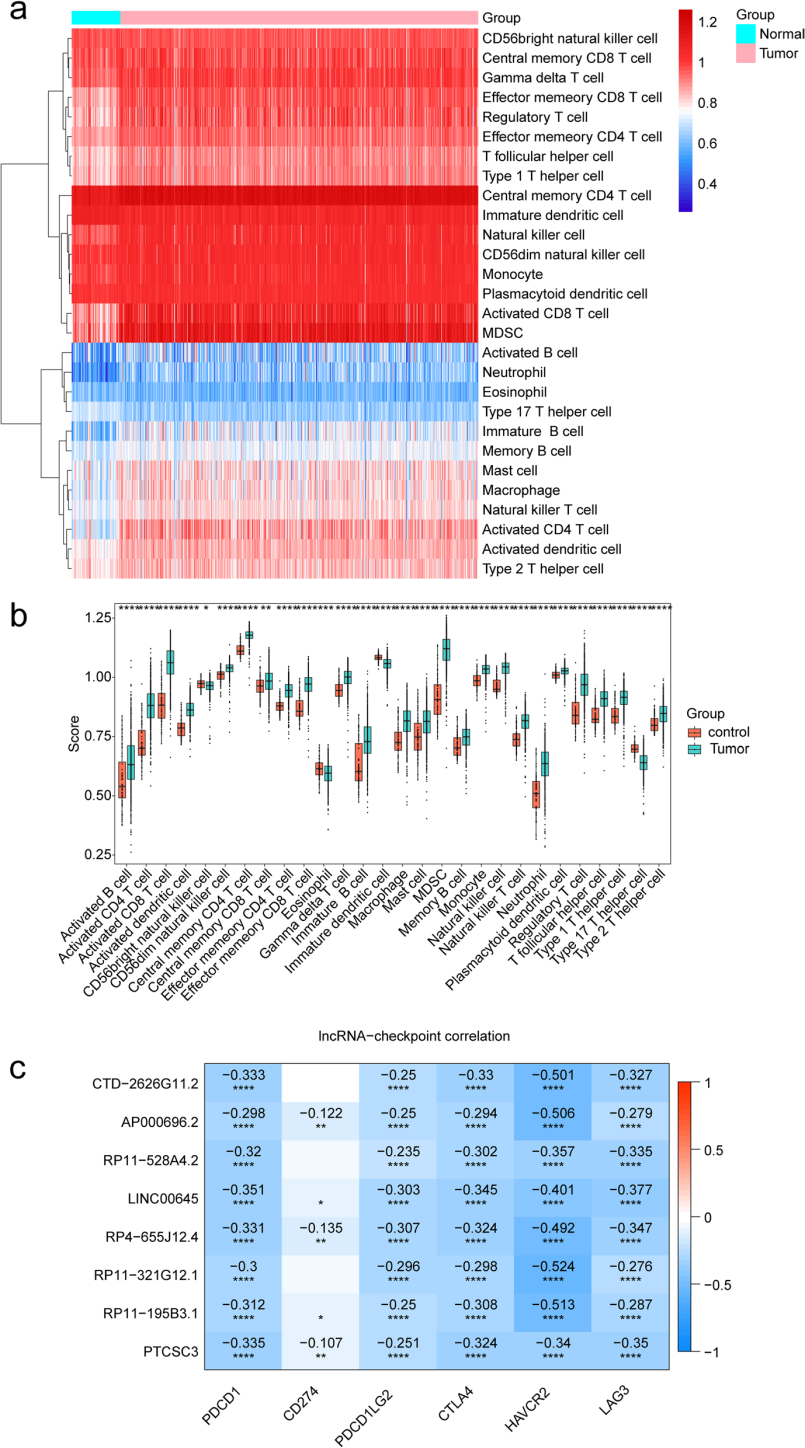
Analysis of immune infiltration in KIRC patient samples and its correlation with hub genes using ssGSEA. **a** Heat map showing the immune scores of 28 immune cells. Red indicates immune cell infiltration and blue indicates suppressed immune cells. **b** Box plot showing immune scores of 28 immune cells in KIRC patients samples and normal samples. **c** The correlation between biomarkers and immune cells

In the TCGA-KIRC dataset, there were significant differences in the infiltration levels of 28 immune cell types between KIRC samples and normal samples (Fig. 8a, b). Among them, the infiltration levels of CD56 (bright) natural killer cells, eosinophils, immature dendritic cells, and helper T cells 17 were significantly lower in KIRC samples compared to normal samples. However, the infiltration levels of the remaining 24 immune cell types in KIRC samples were significantly higher than in normal samples. Furthermore, correlation analysis indicated a negative correlation (*p* < 0.05) between biomarkers and most of the differentially infiltrated immune cells (Fig. 8c).

**Fig. 9:**
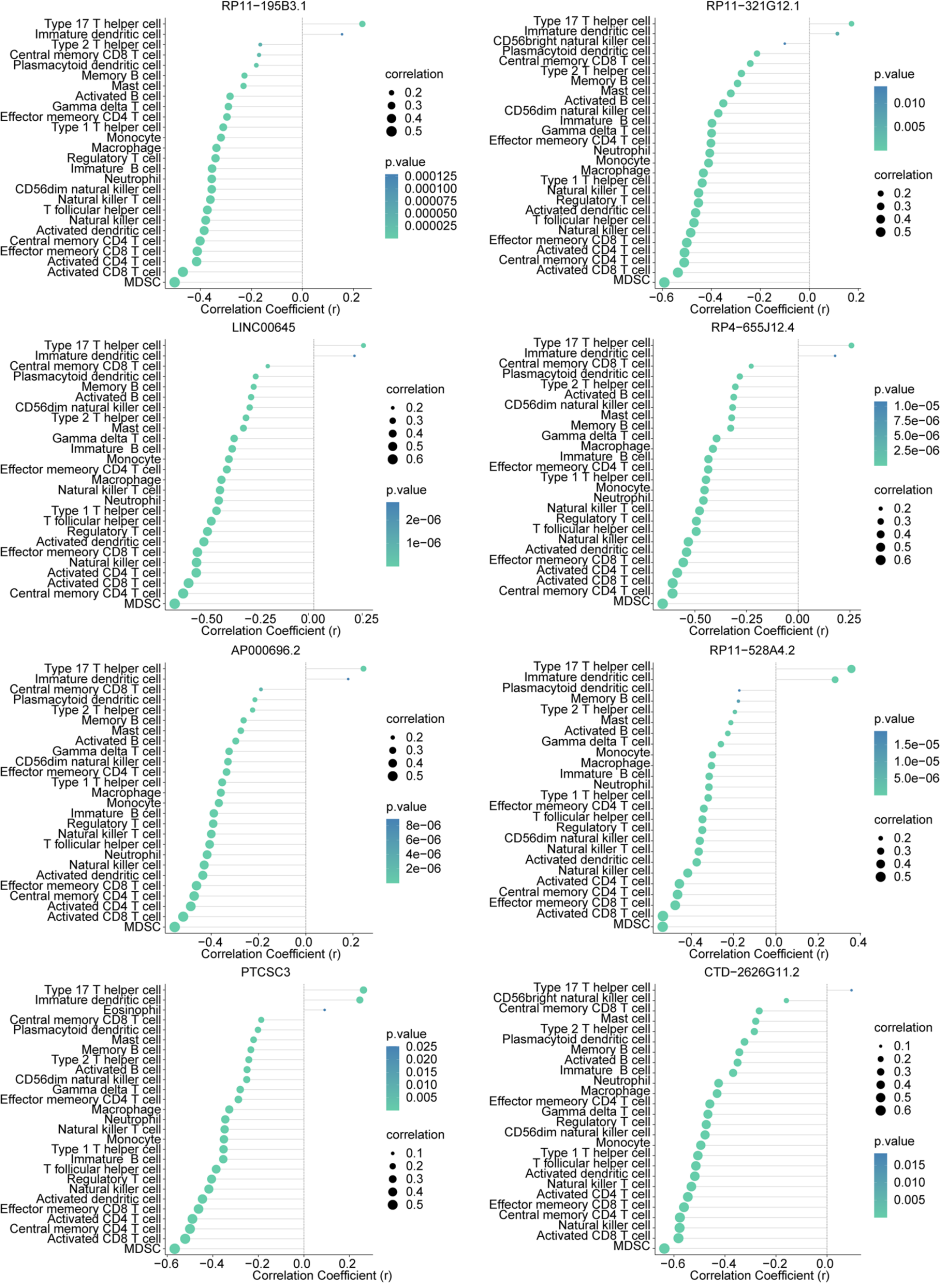
The relevance of biomarkers to common immune checkpoint markers

Additionally, correlation analysis showed a negative correlation (*p* < 0.05) between biomarkers in the TCGA-KIRC dataset samples and common immune checkpoint markers (Fig. 9).

## Discussion

RCC is the sixth most commonly diagnosed cancer in men and the tenth in women worldwide, accounting for 5% and 3% of all cancer diagnoses, respectively [56]. KIRC, being the major pathological subtype, has a higher risk of recurrence, metastasis, and poorer prognosis [57]. RCC is essentially a metabolic disease characterized by reprogramming of energy metabolism, in which patients have segmented metabolic fluxes of glycolysis and, in particular, impaired mitochondrial bioenergetics, oxidative phosphorylation, and lipid metabolism [28–30]. M7G modifications are actively involved in biological and pathological functions by affecting the metabolism of various RNA molecules, processes that are important for normal cellular function and normal development of organisms [18]. This is one of the key reasons why we undertook this study. Moreover, numerous studies indicate that m6A RNA methylation regulators are linked to the development and progress of human cancers [58], and prognostic models based on m6A RNA modification-related lncRNA have improved understanding of KIRC [21, 59]. However, few studies have examined m7G-related lncRNA in predicting KIRC patient prognosis. This study seeks to establish a prognostic model for KIRC patients’ lncRNA to assess its clinical usefulness and explore its correlation with immune cell infiltration and immune checkpoint sites in a systematic manner.

We obtained transcriptome and clinical data of KIRC samples and their corresponding normal controls from TCGA and ICGC databases. Using weighted gene co-expression network analysis, differential analysis, and machine learning, we identified eight differentially expressed m7G-related lncRNAs as biomarkers.

Differential expression analysis revealed that the eight biomarkers were significantly down-regulated in KIRC. LncRNA and m7G correlation analysis showed that all the final genes we obtained were lncRNAs associated with NUDT4. Studies have suggested that NUDT4 is significantly downregulated in various cancers, including KIRC [60–62], which matches our results. Research in other cancer types has also indicated that NUDT4 downregulation affects tumor cell proliferation by impacting the m7G gene [63]. Research indicates that the absence of certain m7G-related regulatory factors is linked to disease. For example, the lack of the METTL1/WDR4 complex impacts tRNA function and translation of multiple mRNAs, leading to abnormal cell cycle progression and proliferation [64]. Thus, combining our results with previously published findings, we propose that downregulation of lncRNA in diseases leads to m7G downregulation, resulting in decreased RNA stability and impaired translation and regulation, ultimately causing the development of various diseases.

Only a few studies have indicated that LINC00645 is downregulated in KIRC, but its role remains unclear [36, 65]. The remaining seven biomarkers have been scarcely reported in KIRC. Our study reports for the first time on the prognostic value of RP4-655J12.4, RP11-321G12.1, RP11-195B3.1, CTD-2626G11.2, AP000696.2, PTCSC3, and RP11-528A4.2 in KIRC. Additionally, previous research has suggested that AP000696.2, LINC00645, and PTCSC3 have potential as biomarkers for different cancers.Additionally, previous research has suggested that AP000696.2, LINC00645, and PTCSC3 have potential as biomarkers for different cancers.AP000696.2 exhibits superior predictive performance compared to traditional tumor markers in esophageal squamous cell carcinoma and may affect patient prognosis and treatment by regulating angiogenesis [66, 67]. LINC00645 is a potential biomarker for acute rejection and graft loss in kidney allografts [68] and was also enriched in the same pathway in our GSEA analysis. Additionally, it can serve as an independent prognostic factor for lung cancer patients [69]. Experimental data suggest that PTCSC3 has antitumor properties, as its overexpression inhibits thyroid papillary carcinoma cell proliferation and development in vitro and in vivo by suppressing glycolysis and promoting PGK1 ubiquitin-mediated degradation [70].

Furthermore, K-M analysis revealed that the expression levels of PTCSC3 and RP11-321G12.1 were significantly associated with the prognosis of KIRC patients. We then used univariate and multivariate Cox regression analyses to examine the association between AP000696.2, PTCSC3, clinical features of KIRC patients, and their prognosis. Based on these prognostic factors, a column chart was constructed to accurately predict the prognosis of KIRC patients.

Pearson correlation analysis was conducted to explore the correlation between the biomarkers and clinical features, as well as their expression levels in different clinical feature subgroups. The results showed that AP000696.2 was positively correlated with patient staging, T-staging, and M-staging in the TCGA-KIRC dataset. Additionally, ROC curves of the biomarkers had an area under the curve greater than 0.95 in both the TCGA-KIRC dataset and the validation set, indicating their potential as diagnostic biomarkers for KIRC by accurately distinguishing KIRC samples from healthy controls.

To identify signaling pathways related to the occurrence and development of KIRC, GSEA was performed on the eight biomarkers. The results showed that these biomarkers were mainly involved in signaling pathways including allograft rejection, cytokine-cytokine receptor interactions, oxidative phosphorylation, chemokine signaling pathway, and other pathways.

Studies have found that certain lncRNAs can serve as potential biomarkers for detecting acute rejection after kidney transplantation [68, 71], providing valuable prognostic information. This suggests that the eight biomarkers identified in our study may have significant prognostic value in KIRC patients.

As early as 2013, the Cancer Genome Atlas research on KIRC found that metabolic changes play a critical role in disease progression, including alterations in the pentose phosphate pathway, fatty acid synthesis pathway, and tricarboxylic acid cycle, which are associated with poor prognosis [72, 73]. Given that oxidative phosphorylation participates in these pathways to provide energy conversion, we speculate that RP4-655J12.4 and CTD-2626G11.2 may affect metabolic pathways through their involvement in oxidative phosphorylation, ultimately leading to poor prognosis for KIRC patients.

Interestingly, the cytokine-cytokine receptor interaction pathway plays a crucial role in adaptive inflammatory host defenses, cell growth, differentiation, cell death, angiogenesis, and developmental and repair processes aimed at restoring homeostasis [74, 75]. This pathway is commonly enriched in the development of liver cancer [76] and colorectal cancer [77]. Studies have shown that chemokines and cytokines, such as TNF-α, IL-2, and chemokine CCL2, play a role in the formation of the cancer microenvironment and are responsible for the migration of inflammatory cells and cancer cells [78].

In addition, we found that LINC00645 plays an oncogenic role in endometrial cancer and glioma with high specificity [79, 80]. It has been reported that LINC00645 can induce the activation of EMT and enhance the migratory and invasive abilities of tumor cells by regulating the expression of miRNA-205-3p and its target gene ZEB1 through the induction of the reverse transforming growth factor TGF-β [81]. These findings provide promising directions for elucidating the potential molecular mechanisms underlying the lncRNA characteristics of KIRC.

The multifunctionality of lncRNAs depends on their subcellular localization. If lncRNAs are located in the cytoplasm, they can act as ceRNAs and regulate mRNA stability or translation [82]. Our research results indicate that most biomarkers are mainly located in the cytoplasm, suggesting that they may participate in post-transcriptional regulatory pathways.

RCC is one of the tumors with the highest degree of immune infiltration in pan-cancer comparisons. Characteristics of the tumor microenvironment heavily influence disease biology and may affect the response to systemic therapy [83]. Several studies have shown that m7G-related genes shape TME by influencing the distribution of immune cells [84–86]. So, we implemented immune infiltration analysis to explore the relationship of immune microenvironment to occurrence and development of KIRC. In our research results, the infiltration levels of 28 immune cells showed significant differences between KIRC samples and normal samples. Immune cells and inflammatory cytokines in the tumor microenvironment can affect tumor development and occurrence. Tumor cells inhibit T cell activation through immune checkpoint, thereby avoiding anti-tumor immune attacks and accelerating tumor deterioration, which is also the main mechanism of cancer immune escape [87]. Therefore, targeting immune checkpoint inhibitors is a significant method for tumor immunotherapy [88]. Our findings indicate changes in the immune microenvironment of KIRC, with most primary immune cells showing significantly higher infiltration levels than normal samples. Additionally, eight biomarkers were found to be significantly negatively correlated with the infiltration levels of immune cells, suggesting their potential involvement in regulating the immune microenvironment of KIRC.

Immunotherapy is mainly represented by immune checkpoint blockade (ICB) and chimeric antigen receptor T cell therapy (CAR-T) [89, 90]. Currently, it is also a first-line treatment for metastatic KIRC [91], with immune checkpoint inhibitors such as PD-1 and CTLA4 being widely used in clinical practice, such as nivolumab and Ipilimumab. ICB has shown significant efficacy in solid tumors, including melanoma, non-small cell lung cancer, and renal cell carcinoma [89]. The upregulation of immune checkpoint molecules, including CTLA-4, PD-1, and PD-L1, has been shown to contribute to tumor immune escape [92]. In addition, some lncRNAs have been found to participate in regulating the expression of PD-1 by affecting the function of specific miRNAs. For example, the interaction between lncRNA SNHG14 and miR-5590-3p upregulates Zinc finger E-box-binding homeobox 1 (ZEB1) to activate the PD-1/PD-L1 immune checkpoint, leading to the inactivation of CD8 + T cells and promoting immune escape of tumor cells in diffuse large B-cell lymphoma [93].

Immune checkpoints are essential predictive indicators for assessing responses to immunotherapy. Our study found that eight lncRNAs exhibited significant negative correlations with some common immune checkpoint sites (PDCD1, PDCD1LG2, CTLA4, HAVCR2, and LAG3) in KIRC. Specifically, RP4-655J12.4, PTCSC3, and AP000696.2 were negatively correlated with CD274. These findings suggest that the eight lncRNA model may play a role in evaluating patients’ response to immune checkpoint blockade therapy. However, there is currently no relevant report explaining the mechanism of action of these eight lncRNAs in immune dysregulation in KIRC.

Although this is not the first time that biomarkers have been predicted for RCC patients based on the transcriptomic data [94], our study for the first time focused primarily on m7G methylation-related lncRNAs and identified eight DE-m7G-lncRNAs that could serve as biomarkers for KIRC. This may provide a new research direction for the study and treatment of KIRC.

## Conclusions

We established a prognostic model for m7G-related lncRNAs using clinical and survival data from a large number of diagnosed KIRC patients in the TCGA database, and validated its prognostic value using an external dataset. However, this study still has some limitations. First, it is a retrospective analysis, which may have selection bias and recall bias. Second, we need more sample datasets for bioinformatics validation, such as CTD-2626G11.2, which was not detected in the ICGC validation set but included in our subsequent analysis for discussion purposes. Finally, further experimental mechanism research and clinical application studies are necessary to verify our findings. We will continue to monitor the progress of related research. In conclusion, our study found that eight lncRNAs may serve as potential biomarkers for KIRC immunotherapy targets.

### Supplementary Information

**Additional file 1: Supplementary Figure 1.** Survival analysis between the high- and low-expression groups of biomarkers. **Supplementary Figure 2.** The expression of biomarkers in KIRC patients of different clinical traits. a: ages; b: N stages; c: M stage. **Supplementary Figure 3.** Receiver operating characteristic curves (ROC) curves in the TCGA-KIRC dataset (a) and the validation dataset (b). AUC, area under the curve.

## Data Availability

The datasets presented in this study can be found and downloaded from online databases, TCGA-KIRC (count data: https://gdc-hub.s3.us-east-1.amazonaws.com/download/TCGA-KIRC.htseq_counts.tsv.gz; fpkm: https://gdc-hub.s3.us-east-1.amazonaws.com/download/TCGA-KIRC.htseq_fpkm.tsv.gz; gene annotation information: https://gdc-hub.s3.us-east-1.amazonaws.com/download/gencode.v22.annotation.gene.probeMap), ICGC-RECU-EU (https://dcc.icgc.org/releases/release_15/RECA-EU/gene_expression.RECA-EU.tsv.gz), ENSEMBL database (https://asia.ensembl.org/index.html), lncLocator (http://www.csbio.sjtu.edu.cn/bioinf/lncLocator/#). Besides, the source code and processed data used for all analyses presented here can be freely obtained from https://github.com/DocotorGuo/prostate-cancer.git.

## Abbreviations

KIRC: Kidney renal clear cell carcinoma
TCGA: The Cancer Genome Atlas
ICGC: International Cancer Genome Consortium
NcRNA: Non-coding RNA
LncRNA: Long Noncoding RNA
m7G: N(7)-methylguanosine
RCC: Renal Cell Carcinoma
TKIs: Tyrosine Kinase Inhibitors
ICBs: Immune Checkpoint Blockers
WGCNA: Weighted Gene Co-Expression Network Analysis
XGBoost: Extreme Gradient Boosting
K-M plot: Kaplan-Meier plot
ROC: Receiver Operating Characteristic Curves
ssGSEA: Single-Sample Gene Set Enrichment Analysis
DCA: Decision Curve Analysis
CIC: Clinical Impact Curve
AUC: Area Under the Curve
CAR-T: Chimeric Antigen Receptor T Cell Therapy
ZEB1: Zinc finger E-box-binding Homeobox 1
MSKCC: Memorial Sloan-Kettering Cancer Center
