# Supplementary material for: Selection of M7G-related lncRNAs in kidney renal clear cell carcinoma and their putative diagnostic and prognostic role
Source: BMC Urol. 2023 Nov 15;23:186. doi: 10.1186/s12894-023-01357-9 (PMC10652602; doi:10.1186/s12894-023-01357-9)
Supplement: Supplementary file 1 — Additional file 1: Supplementary Figure 1. Survival analysis between the high- and low-expression groups of biomarkers. Supplementary Figure 2. The expression of biomarkers in KIRC patients of different clinical traits. a: ages; b: N stages; c: M stage. Supplementary Figure 3. Receiver operating characteristic curves (ROC) curves in the TCGA-KIRC dataset (a) and the validation dataset (b). AUC, area under the curve. [file 12894_2023_1357_MOESM1_ESM.docx]

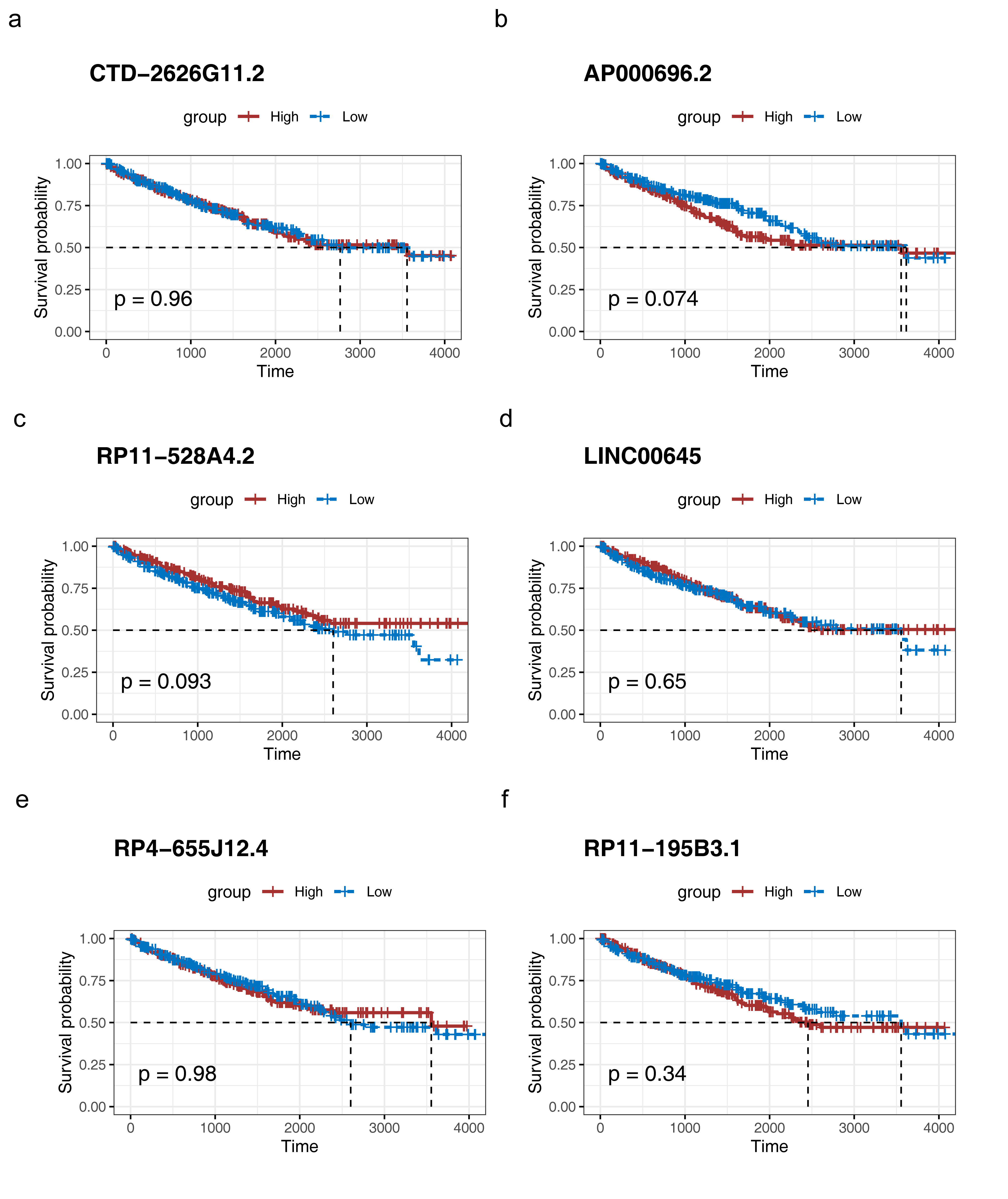


**Supplementary Figure 1** Survival analysis between the high- and low-expression groups of biomarkers.


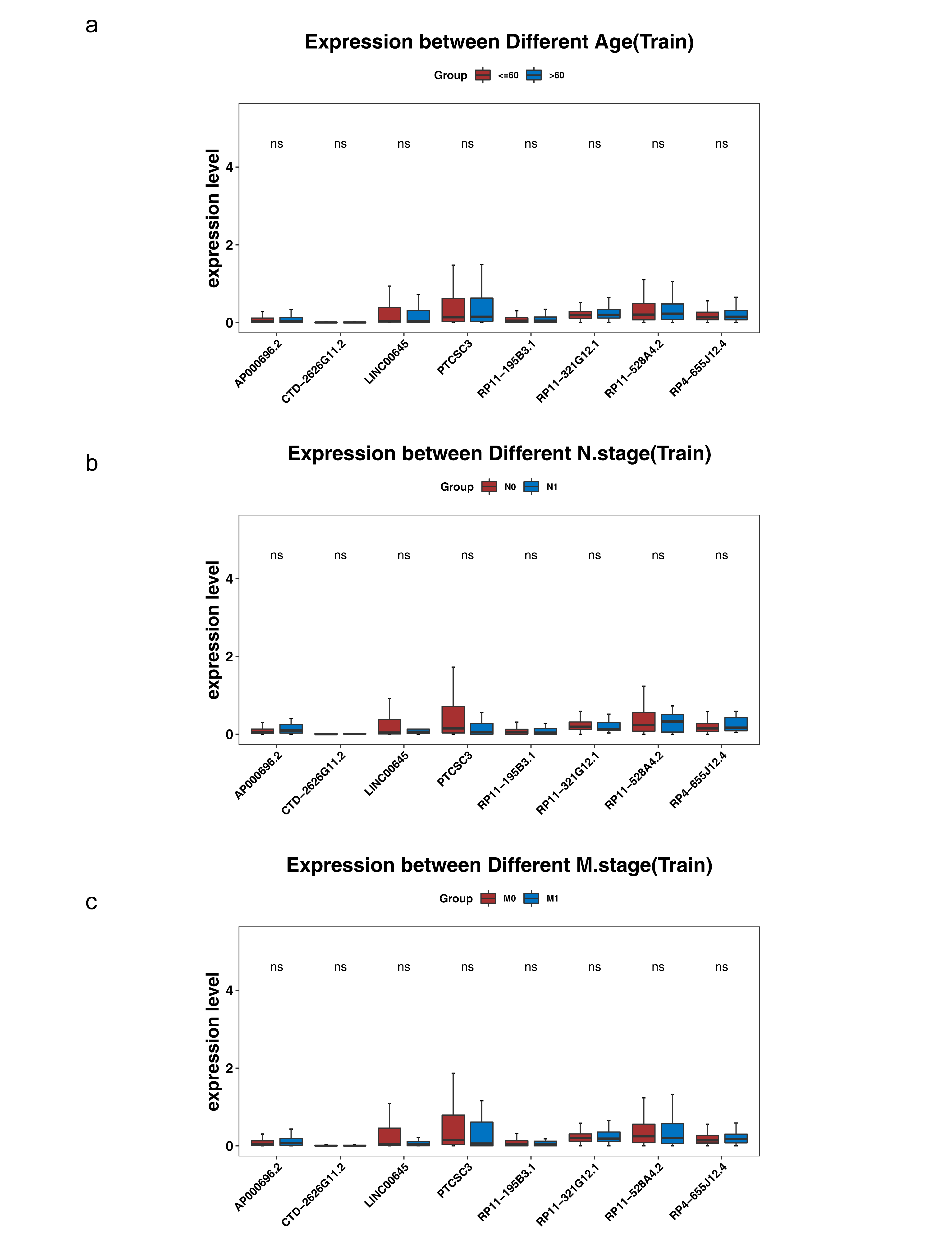


Supplementary Figure 2 The expression of biomarkers in KIRC patients of different clinical traits. a: ages; b: N stages; c: M stage.


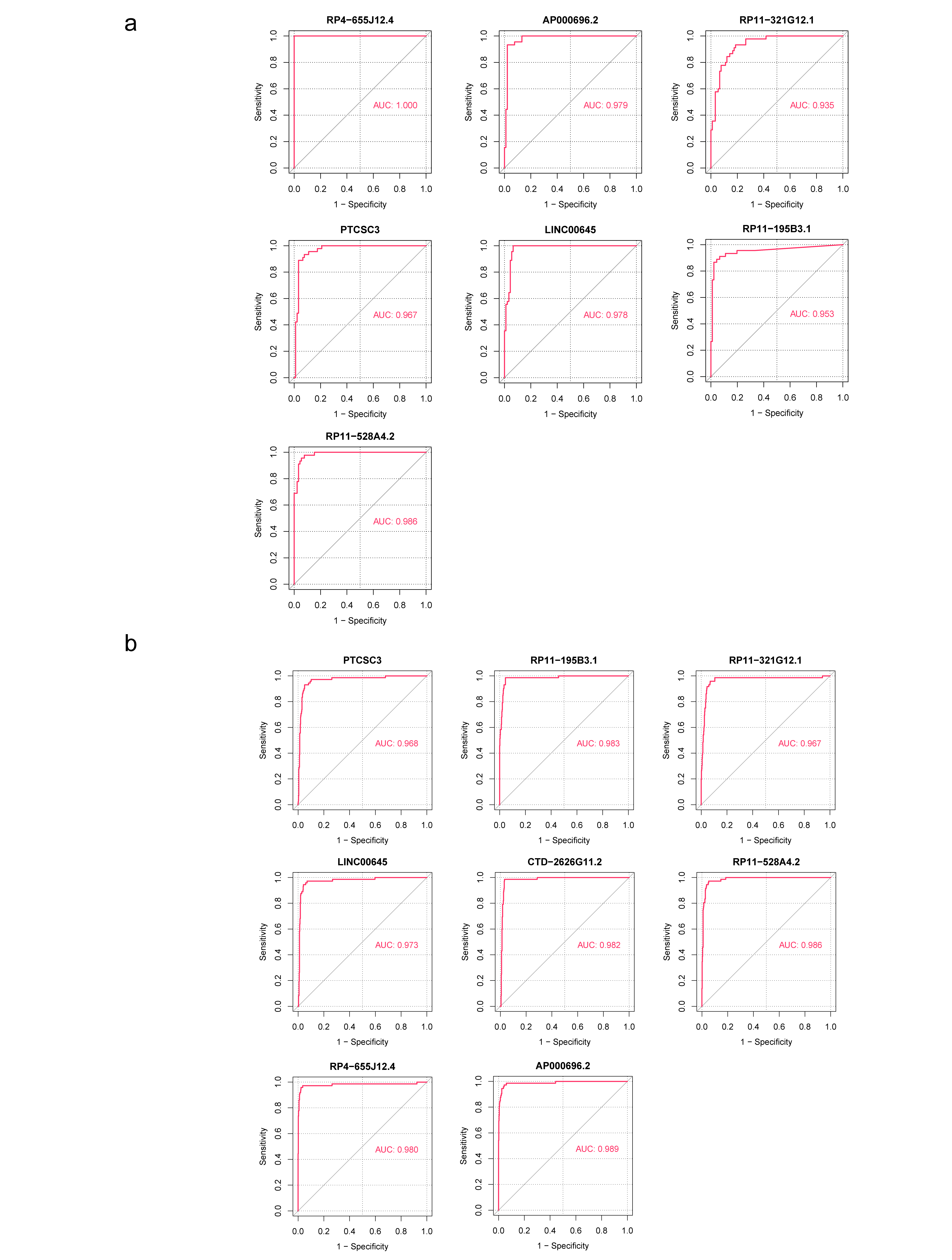
 Supplementary Figure 3 Receiver operating characteristic curves (ROC) curves in the TCGA-KIRC dataset (a) and the validation dataset (b). AUC, area under the curve.
